# *QuickStats:* Percentage* of Residential Care Community Residents with an Advance Directive,^†^ by Census Division^§^ — National Study of Long-Term Care Providers, 2016

**DOI:** 10.15585/mmwr.mm6728a7

**Published:** 2018-07-20

**Figure:**
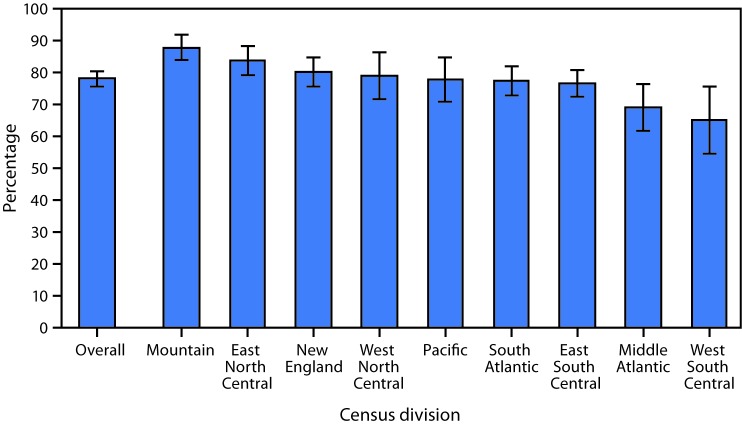
In 2016, 77.9% of residents in residential care communities had an advance directive documented in their files. By Census division, the highest percentage (87.8%) of residents who had an advance directive were located in the Mountain division, followed by residents in East North Central (83.7%), New England (80.0%), West North Central (78.9%), Pacific (77.6%), South Atlantic (77.4%), East South Central (76.4%), Middle Atlantic (68.8%), and West South Central (64.9%).

**Source:** National Center for Health Statistics, National Study of Long-Term Care Providers, 2016. https://www.cdc.gov/nchs/nsltcp/index.htm.

